# Treatment with *Ilex paraguariensis* (yerba mate) aqueous solution prevents hepatic redox imbalance, elevated triglycerides, and microsteatosis in overweight adult rats that were precociously weaned

**DOI:** 10.1590/1414-431X20187342

**Published:** 2018-05-21

**Authors:** E. de Oliveira, N.S. Lima, E.P.S. Conceição, N. Peixoto-Silva, E.G. Moura, P.C. Lisboa

**Affiliations:** Laboratório de Fisiologia Endócrina, Departamento de Ciências Fisiológicas Instituto de Biologia, Universidade do Estado do Rio de Janeiro, Rio de Janeiro, RJ, Brasil

**Keywords:** Early weaning, Programming, Oxidative stress, Steatosis, Liver

## Abstract

Early weaning (EW) leads to overweight, visceral obesity, hyperleptinemia, and insulin resistance in adulthood. Treatment with *Ilex paraguariensis* (yerba mate) improves obesity and insulin resistance in these animals. Here, we evaluated the effects of chronic treatment with yerba mate on the redox balance and liver morphology of overweight early-weaned rats. To induce EW, we wrapped the dams with bandages to interrupt milk access during the last 3 days of lactation. Control pups (C) had free access to maternal milk for the full 21 days of lactation. On postnatal day (PN) 150, EW offspring were subdivided into the EW+YM group, which received the aqueous extract of yerba mate (1 g/kg bw by gavage once a day for 30 days) and the EW group, which received water by gavage for the same period. All rats were euthanized on PN180. The EW group showed higher bound carbonyl (a marker of total protein oxidation), higher TBARS levels (a marker of lipid peroxidation), and lower superoxide dismutase (SOD) activity in liver tissue than the C group, as well as higher triglyceride content and microsteatosis. In plasma, the EW offspring showed higher TBARS levels. One month of yerba mate treatment normalized these parameters. Thus, we have shown evidence that yerba mate improved antioxidant defenses and mitigated liver dysfunction in overweight adult rats that were weaned prematurely.

## Introduction

The growing epidemic of metabolic syndrome has become a great threat to public health not only in developed and developing countries but also in low-income countries. Obesity is the primary causal factor for the development of metabolic syndrome as well as type 2 diabetes, cardiovascular disease, nonalcoholic fatty liver disease, and certain types of cancer (1). Human epidemiological data and experimental animal models have shown that nutritional and environmental changes during the early critical window of life (the intrauterine and/or suckling periods) may affect the physiology of the organism in adulthood as an adaptive mechanism. This phenomenon is known as metabolic programming or developmental plasticity (2–6). Thereby, nutritional and environmental insults during early life favor epigenetic modification of regulatory genes that govern metabolism (7,8).

Rats submitted to early weaning (EW) become obese adults presenting adipocyte hypertrophy, hypothalamic leptin resistance, hypertension, inflammatory profile, high oxidative stress, and liver steatosis (9-11), all of which increase cardiovascular risk. In another model, rats that are overfed during lactation remain overweight in adulthood and present lower antioxidant enzyme activity (catalase, CAT; superoxide dismutase, SOD; and glutathione peroxidase, GPx), along with higher lipid peroxidation in liver and plasma, all of which contribute to impairment of liver insulin signaling and to development of microsteatosis (12). Thus, it seems that oxidative stress may induce liver dysfunction in obese animals, independent of the imprinting factor.

Typical disturbances associated with obesity are influenced by pro-oxidative conditions that can promote injury in various tissues, including liver (13). *Ilex paraguariensis* (yerba mate) has antioxidant properties due to its phenolic compounds, such as caffeoyl derivatives, flavonoids, methylxanthines, tannins, ursolic acid-derived saponins, and vitamins (14). Mate tea (toasted yerba mate) is an herbal infusion obtained from dried leaves of *I. paraguariensis* (15). Originally from the tropical regions of Asia and South America, yerba mate is consumed in Brazil, Paraguay, Argentina, and Uruguay (14). Given its potential health benefits, yerba mate is becoming popular in Europe and North America (15).

Matsumoto et al. (16 [Bibr B17]) reported that acute consumption of roasted yerba mate by healthy women decreased lipid oxidation and increased antioxidant capacity in plasma in addition to increasing the expression of antioxidant enzymes. Furthermore, studies suggest the use of yerba mate antioxidant compounds for obesity management (15–19). Yerba mate improves metabolic syndrome in mice fed a high-fat diet, reversing insulin resistance (20) and reducing body weight, serum cholesterol, triglycerides, and glucose (18). In EW obese adult rats, 30 days of yerba mate treatment normalized abdominal obesity, leptin resistance, and hypertriglyceridemia (21). Therefore, considering that yerba mate has antioxidant potential, we assessed the redox balance in EW obese adult rats treated with yerba mate, as well as its possible role in the prevention of liver steatosis development using tissues as described in a previous publication with the same model (21).

## Material and Methods

### Experimental model

Experiments were conducted following the ethical doctrine of the “three Rs” (reduction, refinement, and replacement) to minimize the number of animals and the suffering caused by the experimental procedures, as prescribed by the principles established in Brazilian Law No. 11.794/2008. The experimental design was approved beforehand by the Animal Care and Use Committee of the Instituto de Biologia, Universidade do Estado do Rio de Janeiro, Rio de Janeiro, RJ, Brazil.

All animals were kept at a controlled temperature (21–23°C) under a fixed light-dark cycle (lights on from 7:00 am to 7:00 pm), with free access to water and food. Three-month-old nulliparous Wistar rats were housed at a ratio of 1 male to 2 females. After mating, twenty pregnant rats were placed in individual cages until the birth of the litters. At birth, each litter was adjusted to 6 male pups per dam to maximize lactation performance.

At the birth of their offspring, lactating rats were randomly divided into two groups. In the control group (C, n=10), the pups had free access to breast milk throughout the lactation period (21 days). For the early weaning group (EW, n=10), at the end of the 17th day of lactation, dams were anaesthetized with a non-lethal dose of thiopental (0.06 mg/100 g bw) and bandaged with tape, physically blocking the litter from accessing breast milk during the last three days of lactation.

Body mass gain and food intake were measured throughout the lives of the rats (21).

### Oral treatment with yerba mate

On postnatal day (PN) 150, two EW offspring from each litter were randomly subdivided into two groups: in EW+yerba mate (EW+YM, n=10), rats received instant yerba mate solution (1 g/kg); in EW + water (EW, n=10), rats received a similar volume of distilled water. One control offspring from each litter was also randomly chosen (n=10) to receive distilled water for the entire treatment period. To prevent isolation stress, we housed two littermates in each cage. Rats received yerba mate solution or distilled water by intragastric gavage once a day for 30 days (21). It is important to note that chronic administration of repeated doses (2 g/kg) of yerba mate extract to rodents does not cause apparent symptoms or signs of toxicity, including changes in behavior, compared with control rats (22).

The yerba mate was prepared from lyophilized instant roasted mate tea, from the same industrial batch (lot A326/06) obtained from Matte Leão¯ (Brazil). A sample of this lot was previously analyzed by our group, and the analysis has been published (21,23). The total phenolic (8.35±0.5 g/L) content was estimated using the Folin-Ciocalteu method. High-performance liquid chromatography (HPLC) analysis of soluble yerba mate powder showed 41.20±8.0 mg/L of chlorogenic acid, 21±4.4 mL/L of caffeine, and 8.57±1.0 mg/L of theobromine; quercetin and rutin were not detected. The roasted mate tea solution was prepared every day before gavage by dissolving the lyophilized powder of yerba mate in filtered water using a homogenizer.

On PN180, rats were fasted for 12 h and then euthanized by decapitation using a rodent guillotine. Blood samples were centrifuged (1000 *g*, 4°C, 20 min) to collect plasma and stored (−20°C) until analysis. The visceral fat (comprising the mesenteric, epididymal and retroperitoneal fat depots) was removed and weighed for evaluation of abdominal adiposity. The liver tissue was collected for the analysis described below.

### Western blotting analysis

Superoxide dismutase 1 (SOD-1), superoxide dismutase 2 (SOD-2), catalase (CAT), glutathione peroxidase (GPx), 4-hydroxynonenal protein adduct (4-HNE), and nitrotyrosine contents in liver tissue were evaluated by western blotting. Liver samples were processed as previously reported (8) in ice-cold lysis buffer (50 mM HEPES, 1 mM MgCl_2_, 10 mM EDTA, Triton X-100 1%, pH 6.4), and protease inhibitor (complete, EDTA free, F. Hoffmann-La Roche Ltd., Switzerland). Homogenates were centrifuged for 5 min (1120 *g*, 4°C). Protein concentrations in the supernatants were determined using the Pierce BCA Protein Assay Kit (Thermo Scientific, USA). Samples (10 μg of total protein) were electrophoresed on 10–12% Tris-glycine sodium dodecyl sulfate polyacrylamide gels. The proteins were transferred to polyvinylidene fluoride membranes (Hybond ECL; Amersham Pharmacia Biotech, UK), which were then blocked in albumin (2%) in TWEEN-20 Tris-buffered saline (T-TBS; 0.02 M Tris/0.15 M NaCl, pH 7.5 containing 0.1% Tween 20) at room temperature for 90 min. Then, the membranes were washed with TBS and incubated with the appropriate primary antibodies (rabbit anti-SOD1 polyclonal antibody, mouse anti-SOD2 monoclonal antibody, mouse anti-CAT monoclonal antibody, mouse anti-Gpx1/2 monoclonal antibody, goat anti-4-HNE polyclonal antibody, and mouse anti-actin monoclonal antibody, each at 1:500 concentration; Santa Cruz Biotechnology, USA) overnight at 4°C. Membranes were washed and incubated with the appropriate secondary antibodies (Santa Cruz Biotechnology) conjugated to HRP at an adequate dilution for 1 h at room temperature. Finally, protein bands were visualized by chemiluminescence (ECL Plus kit, Amersham Biosciences, UK) using an ImageQuant LAS 500 (GE Healthcare, UK). Area and density of the bands were quantified by ImageJ software (Wayne Rasband National Institutes of Health, USA). The results were expressed as percentages relative to the control group.

### Antioxidant enzymes activity

Liver samples (200 mg) were gently homogenized in potassium phosphate buffer with EDTA (Potter homogenizer from Marconi Equipamentos, Brazil). After centrifugation, the homogenates were stored at -80°C until the following analysis. The total protein content was determined by the colorimetric method. Total SOD activity was assayed by measuring the inhibition of adrenaline auto-oxidation as absorbance at 480 nm (24). CAT activity was measured by the rate of decrease in H_2_O_2_ at 240 nm according to the method described by Aebi (25). GPx activity was evaluated according to Flohé and Günzler (26) by measuring the oxidation of NADPH at 340 nm in the presence of H_2_O_2_.

### Measurement of thiobarbituric acid reactive substances (TBARS)

As an index of lipid peroxidation, the yielding of thiobarbituric acid reactive substances (TBARS) during an acid-heating reaction was determined. We added 400 μL of 10% trichloroacetic acid to 200 μL of liver homogenate. These samples were centrifuged (10 min, 3000 *g*, 4°C), and 500 μL of the supernatant was incubated with 500 μL of 0.67% thiobarbituric acid (Sigma Chemical, USA) at 100°C for 30 min. The absorbance at 532 nm was measured with a spectrophotometer.

### Determination of oxidized protein carbonylation

Protein carbonylation was evaluated in liver tissue and plasma as carbonyl groups reacting with 2,4-dinitrophenyl-hydrazine (Sigma) (9). Absorbance was measured at 380 nm, and carbonylation was reported in nmol/mg of protein.

### Triglyceride content

Triglyceride content was determined as previously described by Folch et al. (27). Briefly, 50 mg of liver tissue was homogenized in 1 mL of isopropanol (Vetec, Brazil) and centrifuged (740 *g*, 10 min, 4°C). The triglyceride content of the supernatant was determined with a colorimetric kit following the manufacturer’s instructions (Bioclin, Brazil). The absorbance was measured at 490–540 nm (Hidex, Finland).

### Liver histology

Random liver samples (fragments obtained from all lobes) were fixed in freshly prepared formalin (1.27 mol/L formaldehyde, 0.1 M phosphate-buffered saline, pH 7.2) and embedded in Paraplast Plus¯ (Sigma-Aldrich, USA). Non-serial sections (5 μm) were placed on several glass slides and stained in hematoxylin-eosin. Thus, 10 microscopic fields per animal (n=5) were analyzed at random in a blind analysis, utilizing digital images (TIFF format, 36-bit color, 1360×1024 pixels) acquired with an Olympus DP71 camera and an Olympus BX40 epifluorescence microscope (Olympus, Japan). The evaluation of steatosis was based on the point counting method using a test system composed of 36 test points (P_T_). The volume density (V_V_) was estimated by the following formula: V_V_ [steatosis, liver] = P_P_ [steatosis]/P_T_ [liver], where P_P_ is the number of points located in fat droplets and P_T_ is the total number of points in the test system (28).

### Statistical analysis

Results are reported as means±SE. The GraphPad Prism 4 was used for statistical analyses and graphics (GraphPad Software, Inc., USA). The experimental data were analyzed by one-way ANOVA and Newman-Keuls multiple comparison test. The significance level was set at P<0.05.

## Results

Similar to the results published previously by our group (21), the EW group had no difference in body mass on PN150 (first day of yerba mate treatment). However, on PN180, the EW group had a significant increase in body weight compared with the C group (EW: 461±12 *vs* C: 423±10 g; P<0.05). The EW+YM group presented lower body weight than the EW group (EW+YM: 415±15 *vs* EW: 461±12 g; P<0.05). Adult EW rats exhibited a significant increase in abdominal fat deposition (34%) compared with the C group. The EW+YM rats showed a significant decrease in this parameter (-40% compared with EW rats). In addition, the EW group had higher cumulative food intake than the C group (EW: 23.5±2.7 *vs* C: 19.3±1.8 kg; P<0.05), and the yerba mate normalized the food intake of the EW+YM group compared with the EW group treated with water (EW+YM: 19.28±1.9 *vs* EW: 23.5±2.7 kg; P<0.05).

### Antioxidant enzymes in the liver and plasma

**Figure 1.**
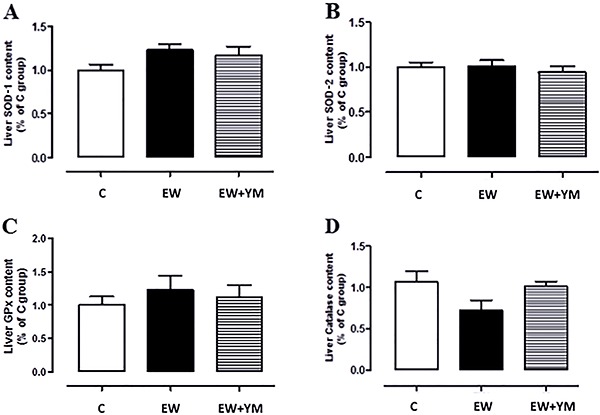
Early weaning and yerba mate treatment did not change liver antioxidant enzymes content. *A*, superoxide dismutase 1 (SOD-1), *B*, superoxide dismutase 2 (SOD-2), *C,* glutathione peroxidase (GPx), and *D*, catalase protein content in the liver at postnatal day 180 of adult rats that were normally breastfed for 21 days (C), early weaned (EW), or EW that received yerba mate for 30 days (EW+YM). Data are reported as means±SE of 10 rats per group (ANOVA).

We did not find any changes in liver protein content of the antioxidant enzymes SOD-1, SOD-2, GPx, and CAT among the three groups (Figure 1).

**Figure 2.**
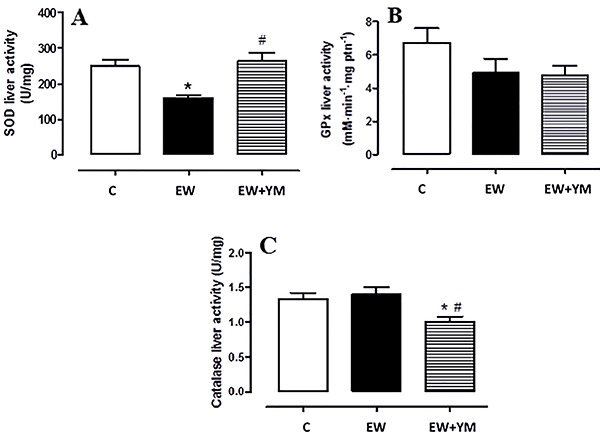
Early weaning decreased superoxide dismutase (SOD) activity, and yerba mate treatment reduced catalase activity in the liver. *A*, SOD, *B*, glutathione peroxidase (GPx), and *C*, catalase activities in the liver at postnatal day 180 of adult rats that were normally breastfed for 21 days (C), early weaned (EW), or EW that received yerba mate for 30 days (EW+YM). Data are reported as means±SE of 10 rats per group. *P<0.05 *vs* C; ^#^P<0.05 *vs* EW (ANOVA and Newman-Keuls multiple comparison test).

Regarding enzyme activity in the liver, we found lower SOD activity in the EW group (-36%, P<0.05, Figure 2A), which was reversed by treatment with yerba mate. GPx was unchanged across groups (Figure 2B). The yerba mate intervention decreased CAT activity in the EW+YM group compared with the C and EW groups (-25 and -29%, respectively, P<0.05, Figure 2C).

**Figure 3.**
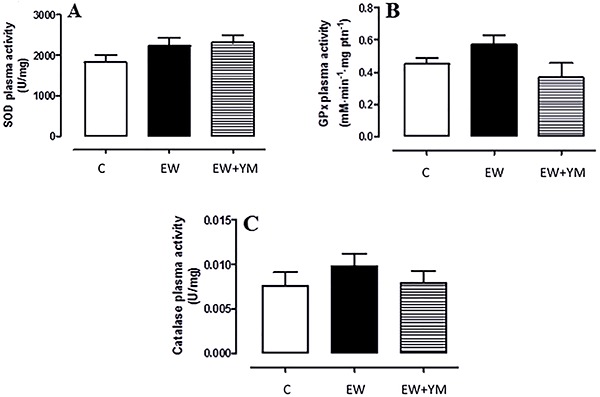
Early weaning and yerba mate treatment did not alter plasma antioxidant enzymes activities. *A*, superoxide dismutase (SOD), *B*, glutathione peroxidase (GPx), and *C*, catalase activities in the plasma at postnatal day 180 of adult rats that were normally breastfed for 21 days (C), early weaned (EW), or EW that received yerba mate for 30 days (EW+YM). Data are reported as means±SE of 10 rats per group (ANOVA).

In plasma, there was no difference among the three groups in antioxidant enzymes activity (Figure 3).

### Lipid peroxidation and protein oxidation in the liver and plasma

**Figure 4.**
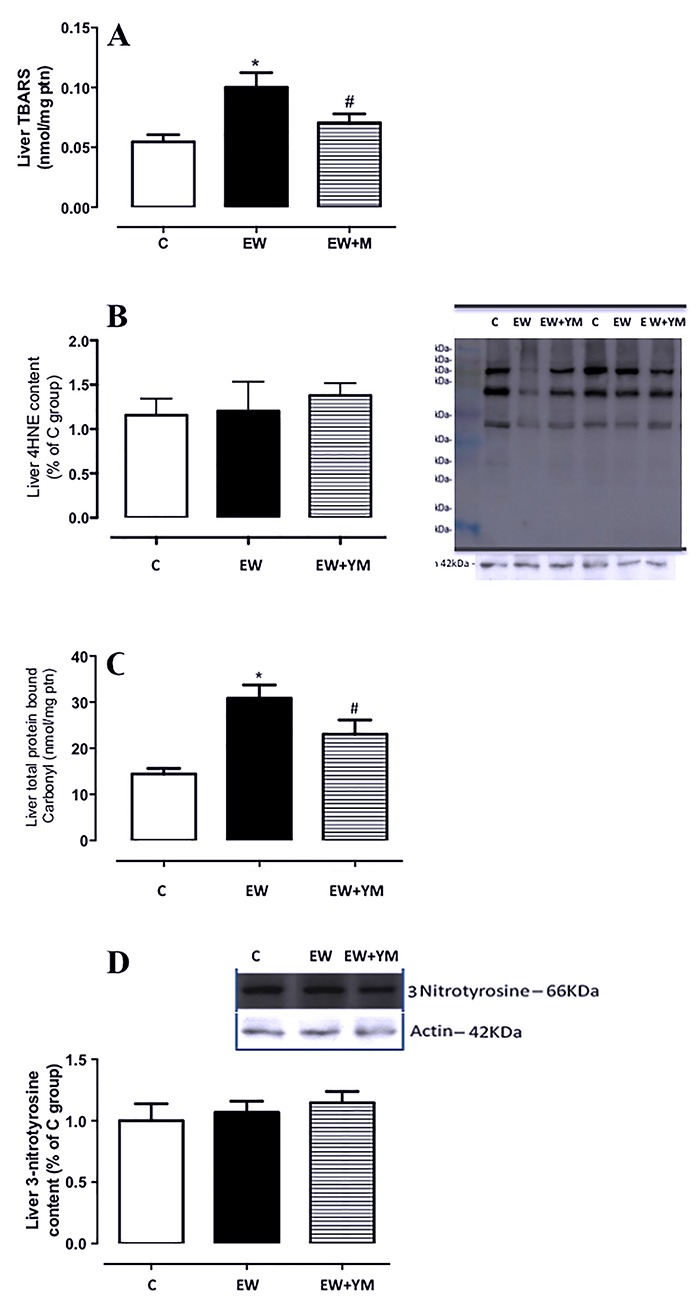
Yerba mate treatment prevented the increase of thiobarbituric acid reactive substances (TBARS) and total protein bound carbonyl in the liver caused by early weaning. *A*, TBARS content, *B,* 4-hydroxynonenal (4HNE) protein adducts, *C*, total protein bound carbonyl, and *D*, 3-nitrotyrosine in the liver at postnatal day 180 of adult rats that were normally breastfed for 21 days (C), early weaned (EW), or EW that received yerba mate for 30 days (EW+YM). Data are reported as means± SE of 10 rats per group. *P<0.05 *vs* C; ^#^P*<*0.05 *vs* EW (ANOVA and Newman-Keuls multiple comparison test).

In the liver, EW rats showed elevated TBARS concentrations (Figure 4A: +87%, P<0.05), and treatment with yerba mate reversed this parameter. The EW group also showed an increase in total carbonylated protein (+114%, P<0.05, Figure 4C), which was partially reversed by treatment with yerba mate (-25% *vs* EW, P<0.05).

There was no difference among the groups in liver 4-HNE or 3-nitrotyrosine content (Figures 4B and D).

**Figure 5.**
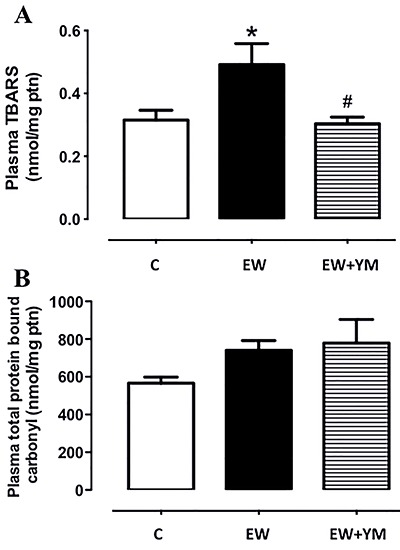
Yerba mate treatment prevented the increase of plasma thiobarbituric acid reactive substances (TBARS) levels caused by early weaning but early weaning and yerba mate treatment did not change plasma protein bound carbonyl. *A*, TBARS content and *B*, total protein bound carbonyl in the plasma at postnatal day 180 of adult rats that were normally breastfed for 21 days (C), early-weaned (EW), or EW that received yerba mate for 30 days (EW+YM). Data are reported as means±SE of 10 rats per group. *P<0.05 *vs* C; ^#^P*<*0.05 *vs* EW (ANOVA and Newman-Keuls multiple comparison test).

EW rats showed elevated TBARS concentration in plasma (Figure 5A: +56%, P<0.05), and treatment with yerba mate reversed this parameter. There was no difference between the groups in plasma levels of total carbonylated protein (Figure 5B).

### Liver triglyceride content and morphology

**Figure 6.**
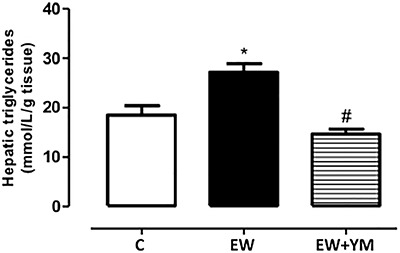
Yerba mate treatment prevented the increase of liver triglycerides induced by early weaning. Liver triglycerides content at postnatal day 180 of adult rats that were normally breastfed for 21 days (C), early weaned (EW), or EW that received yerba mate for 30 days (EW+YM). Data are reported as means±SE of 10 rats per group. *P<0.05 *vs* C; ^#^P*<*0.05 *vs* EW (ANOVA and Newman-Keuls multiple comparison test).

Liver triglyceride content was higher in the EW group than in the C group (+47%, P<0.05, Figure 6). The EW+ YM group had lower liver triglyceride content than the EW group (-46%, P<0.05, Figure 6).

**Figure 7.**
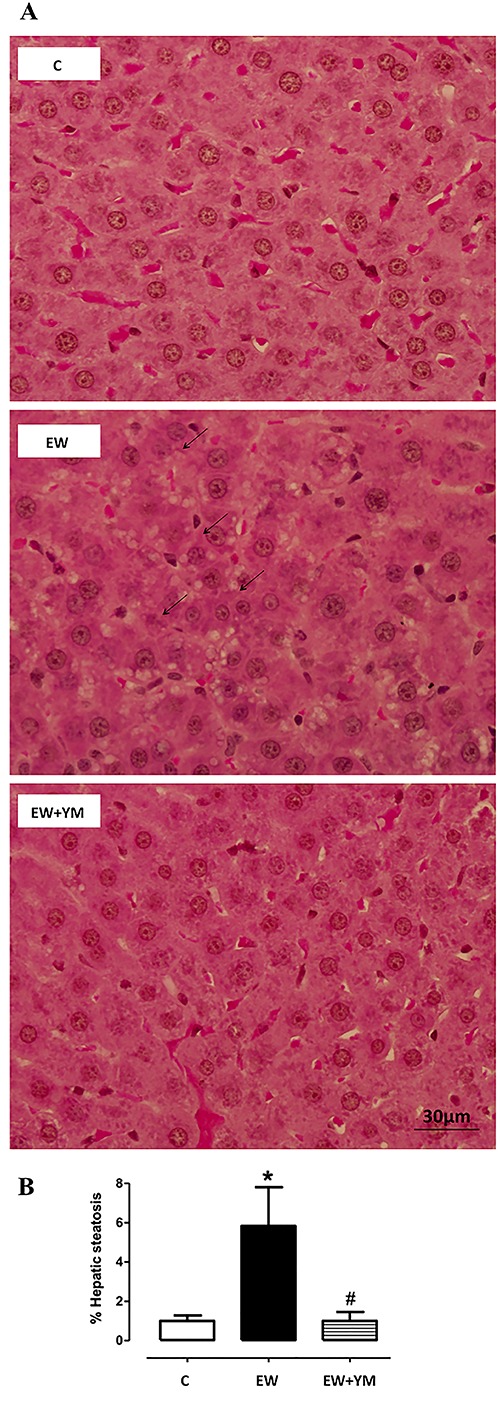
Yerba mate treatment prevented liver fat accumulation induced by early weaning. *A*, Photomicrographs of liver tissue stained with hematoxylin-eosin with same magnification (magnification 60×; bar: 30 μm). *B*, percentage steatosis at postnatal day 180 of adult rats that were normally breastfed for 21 days (C), early weaned (EW), or EW that received yerba mate for 30 days (EW+YM). The arrows indicate the droplets of lipids in the hepatocytes characterizing a microsteatosis. Data are reported as means±SE of 5 rats per group. *P<0.05 *vs* C; ^#^P<0.05 *vs* EW (ANOVA and Newman-Keuls multiple comparison test).

As depicted in Figure 7, EW rats presented steatosis, characterized by drops of lipids in cytoplasm (4.8-fold increase, P<0.05, Figure 7B), and treatment with yerba mate aqueous extract for 30 days prevented this fat accumulation, restoring values similar to those of the control group.

## Discussion

Yerba mate is a beverage that contains several antioxidant compounds, which can have different effects when used in combination than when given separately. In other words, isolation of these compounds may display effects that do not reflect the effect of the complete yerba mate aqueous solution. In a previous study, we showed that treatment with yerba mate for 30 days minimized body mass gain, adiposity, and insulin resistance in obese rats programmed by early weaning (21). Therefore, our objective in the present study was to evaluate the effects of this treatment upon the liver morphology and oxidative stress parameters in this programming model of obesity.

Obesity promotes deregulated production of adipokines and a high flux of free fatty acids in the circulation through the mechanism of lipolysis, demonstrating the establishment of a vicious cycle with insulin resistance. In this sense, the beneficial alterations promoted by yerba mate in the body composition of the EW+YM group may be related to the improvement in hepatic dysfunction. A recent study showed that mice receiving a high fat diet with supplemental yerba mate (added in the chow) showed reduction of hepatic steatosis mainly due to recovery of insulin sensitivity. These mice had increased expression of thermogenic proteins (UCP1 and CIDEA), which favors the increase of energy expenditure, improvement of dyslipidemia due to increased fecal excretion of lipids, and reduction of *de novo* lipogenesis enzymes. All changes were justified by the effects of yerba mate on insulin sensitivity (29). Another study showed that treatment of obese mice with yerba mate for seven weeks also improved liver steatosis and increased peripheral insulin sensitivity, in addition to promoting a reduction of adipocyte proliferation (20).

Obesity is often characterized by high oxidative stress resulting from an imbalance between the cellular production of reactive oxygen species and their inefficient neutralization by antioxidant defenses. This relationship can be observed in another programming model, in which rats are submitted to neonatal overnutrition by the reduction of litter size. In adulthood, these rats presented overweight, high visceral adiposity, insulin resistance in the liver, diminished SOD, CAT, and GPx activity in the liver, and elevated TBARS levels in the liver and plasma, indicating higher oxidative stress. All these metabolic changes contribute to liver injuries, such as the development of steatosis (12). Treatment with yerba mate for four weeks normalized liver antioxidant enzyme activity, and reduced lipid peroxidation and liver steatosis in adult rats overfed during nursing (30).

The antioxidants in yerba mate, especially caffeic and chlorogenic acids, can contribute to protective effects against obesity-related oxidative damage. Indeed, chlorogenic acid suppresses oxidative stress caused by hepatic lipid peroxidation in rats submitted to ischemia and reperfusion injury (31). Caffeic acid is a product of chlorogenic acid hydrolysis in the small intestine. Both caffeic and chlorogenic acids were associated with lower triglyceride (in plasma, liver, and heart) and cholesterol concentrations (in plasma, adipose tissue, and heart) in obese mice (32). Treatment with caffeic acid decreases free radical production and GPx content in rats with drug-induced oxidative stress (33). Based on these findings, it could be postulated that the effects of yerba mate on oxidative stress and microsteatosis can be partially attributed to its chlorogenic-acid-related products. Recently, it was demonstrated *in vitro* that chlorogenic acid was able to restore the oxidant/antioxidant status in hepatocytes from Wistar rats exposed to oxidative stress. Additionally, pretreatment with chlorogenic acid rendered hepatocytes resistant to oxidative conditions and promoted maintenance of cellular homeostasis (34).

In the present study, the hepatic changes observed in the EW rats were characterized by a reduction of SOD activity and an increase in carbonylated proteins and TBARS, which might be responsible for microsteatosis, a cause of nonalcoholic fatty liver disease, in the non-treated EW rats. In the EW+YM group, 30 days of treatment with yerba mate aqueous solution normalized all these parameters. Our data corroborate with that of Gao et al. (35), who showed that yerba mate increases plasma SOD and decreases TBARS levels in hyperlipidemic hamsters. Another study showed that treatment with yerba mate (0.5, 1.0, or 2.0 g/kg) for 60 days improves antioxidant defense and decreases peroxidative damage in the mouse liver (36).

Taken together, the current observations reinforce the concept that early weaning is a risk factor for later obesity and several comorbidities, such as oxidative stress and liver microsteatosis. Yerba mate treatment may be helpful in the control of metabolic disturbances in obesity, and our findings indicate its potential importance in the future as a therapeutic tool to prevent damage caused by redox imbalance. Isolated yerba mate components may also improve the aforementioned metabolic alterations, and we are developing new experiments using these compounds so that, beyond the effects of yerba mate, we can better assess the effects of its isolated components on liver dysfunction *in vivo* in EW offspring. Furthermore, early exposure to antioxidant conditions might prevent liver dysfunction and the development of obesity. Therefore, experimental studies must be designed to elucidate whether the early prevention of oxidative stress might be a good strategy to avoid future metabolic diseases.
